# Post-traumatic disorders and psychosis in migrant offenders: symptom overlap or comorbidity?

**DOI:** 10.1192/j.eurpsy.2026.10376

**Published:** 2026-07-31

**Authors:** F. Boaron

**Affiliations:** Dipartimento ad attività integrata Salute Mentale e Dipendenze Patologiche, AUSL Reggio Emilia - Italy, Reggio Emilia, Italy

## Abstract

**Abstract:**

There is broad consensus that exposure to early traumatic events constitutes a transdiagnostic risk factor for adverse physical, social, and mental health outcomes. The landmark CDC-Kaiser Permanente Adverse Childhood Experiences (ACEs) study demonstrated a clear dose–response relationship between childhood adversity and increased risk of psychosis, mood disorders, substance use disorders, cardiovascular disease, diabetes mellitus, early pregnancy, school dropout, and unemployment. Subsequent meta-analyses and longitudinal studies have further established ACEs as a powerful predictor of antisocial behaviours.

Clinical observations conducted in the Italian REMS (Residences for the Execution of Security Measures), psychiatric facilities dedicated to the treatment of offenders with mental disorders, are fully consistent with these findings. In an observational study of a small sample (N = 38) of patients admitted to the Bologna REMS, the prevalence of ACEs exceeded 90%, with 18% of individuals exposed to four or more distinct categories of childhood adversity. This subgroup shows high cumulative trauma burden, persistent psychotic symptoms and behavioural dysregulation despite adequate psychopharmacological treatment, resulting in prolonged institutionalisation. In contrast, trauma load was not significantly associated with either the type or the severity of the index offence. In some of these cases, the implementation of a trauma-focused therapeutic approach led to marked clinical improvement and subsequent discharge to non-custodial residential facilities.

These findings are particularly relevant in migrant populations, who are frequently exposed to complex and repeated traumatic experiences across the lifespan, including pre-migration trauma, trauma during the migration journey, and post-migration factors such as social exclusion and marginalisation.

The high prevalence of ACEs observed in samples of patients with antipsychotic-resistant psychosis raises a critical clinical and nosographic issue: in a non-negligible proportion of cases, symptoms conventionally interpreted as the primary expression of a psychotic disorder may instead reflect trauma-related psychopathological processes, including complex post-traumatic symptomatology or true comorbidity between complex PTSD (PTSD-C) and psychosis. These observations have direct implications for differential diagnosis and treatment planning, underscoring the need for systematic trauma assessment and the integration of trauma-focused interventions also within secure psychiatric settings.

**Disclosure of Interest:**

None Declared

